# 慢性阻塞性肺疾病对非小细胞肺癌术后复发风险的影响

**DOI:** 10.3779/j.issn.1009-3419.2018.03.21

**Published:** 2018-03-20

**Authors:** 光亮 强, 其多 余, 朝阳 梁, 之乙 宋, 彬 石, 永庆 郭, 德若 刘

**Affiliations:** 100029 北京，中日友好医院胸外科 Department of Thoracic Surgery, China-Japan Friendship Hospital, Beijing 100029, China

**Keywords:** 肺肿瘤, 慢性阻塞性肺疾病, 手术, 肺功能, 预后, Lung neoplasms, Chronic obstructive pulmonary disease, Surgery, Pulmonary function, Prognosis

## Abstract

**背景与目的:**

肺癌和慢性阻塞性肺病（chronic obstructive pulmonary disease, COPD）和非小细胞肺癌（non-small cell lung cancer, NSCLC）均是呼吸系统的常见病和多发病，在世界范围内均是高发病率和高死亡率的疾病。本研究旨在探讨COPD的严重程度是否影响NSCLC切除术后的远期生存。

**方法:**

回顾性分析421例肺叶切除术的NSCLC患者资料，根据慢性阻塞性肺疾病全球倡议（Global Initiative for Chronic Obstructive Lung Disease, GOLD）指南对COPD患者严重程度进行分级，将入组患者分为3组，比较临床病理特征和无复发生存（recurrence-free survival, RFS）的差异。

**结果:**

合并COPD者共172例，其中轻度124例，中度46例，重度2例。随着COPD严重程度增加，术后复发率增加（*P* < 0.001）。无COPD组、轻度COPD组（GOLD-1）和中重度COPD组（GOLD-2/3）的5年无复发生存率分别为78.1%，70.4%和46.4%，差异有统计学意义（*P* < 0.001）。单因素分析结果显示，年龄、性别、吸烟史、COPD严重程度、肿瘤直径、组织学类型和病理分期是影响患者无复发生存的危险因素。Cox多因素回归分析结果显示，年龄、性别、中重度COPD和病理分期是影响患者预后的独立危险因素。

**结论:**

合并中重度COPD是影响NSCLC患者术后无复发生存的独立危险因素，可结合患者术前肺功能来判断预后，更准确地预测复发风险，为高危患者制定合理的个体化治疗方案。

## Abstract (en)

**Background and objective:**

Lung cancer and chronic obstructive pulmonary disease (COPD) are both common diseases in respiratory system and the leading causes of deaths worldwide. The purpose of this study was to determine whether the severity of COPD affects long-term survival in non-small cell lung cancer (NSCLC) patients after surgical resection.

**Methods:**

A retrospective research was performed on 421 consecutive patients who had undergone lobectomy for NSCLC. Classification of COPD severity was based on guidelines of the Global Initiative for Chronic Obstructive Lung Disease (GOLD). Characteristics among the three subgroups were compared and recurrence-free survivals were analyzed.

**Results:**

A total of 172 patients were diagnosed with COPD, 124 as mild (GOLD-1), 46 as moderate(GOLD-2), and 2 as severe (GOLD-3). The frequencies of recurrence were significantly higher in higher COPD grades group (*P* < 0.001). Recurrence-free survival at five years were 78.1%, 70.4%, and 46.4% in Non-COPD, GOLD-1 COPD, and GOLD-2/3 COPD groups, respectively (*P* < 0.001). In univariate analysis, age, gender, smoking history, COPD severity, tumor size, histology and pathological stage were associated with recurrence-free survival. Multivariate analyses showed that older age, male, GOLD-2/3 COPD, and advanced stage were independent risk factors associated with recurrence-free survival.

**Conclusions:**

NSCLC patients with COPD are at higher risk for postoperative recurrence, and moderate/severe COPD is an independent unfavorable prognostic factor. The severity of COPD based on pulmonary function test can be a useful indicator to identify patients at high risk for recurrence. Therefore, it can contribute to adequate selection of the appropriate individualized treatment.

肺癌和慢性阻塞性肺病（chronic obstructive pulmonary disease, COPD）是呼吸系统的常见病和多发病，在世界范围内均是高发病率和高死亡率的疾病。肺癌目前居恶性肿瘤死亡率第一位，COPD的发病率、住院率、死亡率也呈上升趋势，占当今全球死亡原因的第四位，预计2020年将上升至第三位^[1]^。过去多年来将COPD和肺癌作为两个独立的疾病进行研究，近年来流行病学调查发现COPD患者中肺癌发病率是一般人群的4.5倍^[2, 3]^，而40%-70%的肺癌患者合并COPD^[4]^，两者之间存在一定的关联性。COPD和肺癌在病因学上具有一些共同的危险因素，如吸烟、男性、高龄、遗传易感性、职业和环境因素等；在发病机制上存在共同的慢性炎症反应、免疫反应等。COPD患者因反复肺部感染进而导致呼吸系统慢性炎症以及肺功能持续性性下降，合并COPD的肺癌患者手术风险和术后并发症发生率增加，围手术期易发生肺部感染、肺不张、呼吸衰竭、肺漏气时间延长等。但合并COPD是否影响肺癌患者术后的远期生存尚不明确，本研究旨在探讨COPD对非小细胞肺癌（non-small cell lung cancer, NSCLC）完全切除术后复发风险的影响。

## 1 资料和方法

### 1.1 研究对象

回顾性分析2012年1月-2015年6月在我院接受胸腔镜肺叶切除、系统性纵隔淋巴结清扫手术治疗的NSCLC患者。根据世界卫生组织（World Health Organization, WHO）2004年肺肿瘤分类和国际肺癌研究协会（International Association for the Study of Lung Cancer, IASLC）2009年第7版TNM分期标准进行病理分型和术后分期。纳入标准为：（1）病理学检查确定为原发性非小细胞肺癌；（2）术前1周内进行肺功能检测；（3）临床病理资料完整。排除标准为：（1）既往曾患其他恶性肿瘤；（2）术前接受过放疗、化疗等诱导治疗；（3）术前合并肺炎、肺不张、肺间质纤维化及其他可能影响肺功能指标者；（4）原发肿瘤未完全切除，肉眼或镜下切缘阳性者；（5）术后30天内出现严重并发症或死亡。

### 1.2 肺功能测定方法

术前1周内对所有患者行肺功能检测，采用德国Jaeger Master Screen自动肺功能检测仪对所有受试者安静后测定基础肺功能。测定项目包括用力肺活量（forced vital capacity, FVC）、用力肺活量占预计值百分比（FVC%）、第1秒用力呼气容积（forced expiratory volume in one second, FEV_1_）、第1秒用力呼气容积占预计值百分比（FEV_1_%）、第1秒用力呼气容积占用力肺活量百分比（FEV_1_/FVC%）。根据肺功能测定结果，按2015年慢性阻塞性肺疾病全球倡议（Global Initiative for Chronic Obstructive Lung Disease, GOLD）的诊断标准^[5]^：FEV_1_/FVC% < 70%诊断为COPD。COPD严重程度分级以FEV_1_的降低程度来确定：FEV_1_≥80%预计值为轻度，50%≤FEV_1_ < 80%预计值为中度，30%≤FEV_1_ < 50%预计值为重度，FEV_1_ < 30%预计值为极重度。

### 1.3 观察指标

术后通过门诊或住院复查、信件和电话等形式完成随访，随访截止时间为2017年6月。术后2年内每3个月复查一次，2年后每6个月复查一次，项目包括血清肿瘤标记物、胸部CT、头部CT或MR、腹部CT或B超，全身骨扫描。复发包括胸腔内局部复发和胸腔外转移性复发，复发时间以初次确定复发病灶为准。观察起点为手术日，终点为肿瘤复发或死亡，无复发生存时间（recurrence-free survival, RFS）定义为手术日至观察终点或随访截止时间。所有患者均获有效随访，随访时间为10个月-83个月，中位随访时间为50个月。

### 1.4 统计学方法

所有资料采用SPSS 21.0软件进行统计学分析，计数资料用例数及百分比表示，组间比较采用独立样本的卡方检验或*Fisher*确切概率法检验；计量资料采用均数±标准差（Mean±SD）表示，组间比较采用采用单因素方差分析，相关性分析采用*Spearman*秩相关检验。*Kaplan-Meier*法绘制生存曲线，*Log-rank*检验统计学差异。单因素分析中差异有统计学意义的危险因素纳入*Cox*比例风险回归模型进行多因素分析。*P* < 0.05为差异有统计学意义。

## 2 结果

### 2.1 基本资料

共纳入421例患者资料，平均年龄67岁（32岁-84岁），男性260例，女性161例，有吸烟史者273例，伴COPD者172例，其中轻度124例，中度46例，重度2例。TNM病理分期Ⅰ期356例，Ⅱ期36例，Ⅲa期29例。根据GOLD分级标准，将全部患者根据COPD严重程度分为无COPD组（Non-COPD）249例、轻度COPD组（GOLD-1）124例、中重度COPD组48例（GOLD-2 46例，GOLD-3 2例），组间比较结果见表 1。随着COPD严重程度增加，高龄、男性、吸烟、非腺癌的比例增加，病理分期和术后复发率也增加，差异有统计学意义（*P* < 0.05）。

**1:** COPD严重程度不同的NSCLC患者临床病理特征比较 Patient clinicopathological characteristics depending on the severity of chronic obstructive pulmonary disease (COPD)

Factor	Non-COPD (*n*=249)	Mild COPD (*n*=124)	Moderate/severe COPD (*n*=48)	*P*
Age (yr)				< 0.001
< 65	118 (47.4%)	24 (19.4%)	13 (27.1%)	
≥65	131 (52.6%)	100 (80.6%)	35 (72.9%)	
Gender				< 0.001
Female	117 (47.0%)	35 (28.2%)	9 (18.8%)	
Male	132 (53.0%)	89 (71.8%)	39 (81.3%)	
Smoking history				< 0.001
No	111 (44.6%)	33 (26.6%)	4 (8.3%)	
Yes	138 (55.4%)	91 (73.4%)	44 (91.7%)	
Tumor size (cm)				0.405
≤3	214 (85.9%)	100 (80.6%)	41 (85.4%)	
> 3	35 (14.1%)	24 (19.4%)	7 (14.6%)	
Tumor location				0.289
Left upper lobe	65 (26.1%)	41 (33.1%)	8 (16.7%)	
Left lower lobe	37 (14.9%)	16 (12.9%)	8 (16.7%)	
Right upper lobe	83 (33.3%)	35 (28.2%)	14 (29.2%)	
Right middle lobe	18 (7.2%)	9 (7.3%)	2 (4.2%)	
Right lower lobe	46 (18.5%)	23 (18.5%)	16 (33.3%)	
Histology				< 0.001
Adenocarcinoma	213 (85.5%)	78 (62.9%)	27 (56.3%)	
Non-adenocarcinoma	36 (14.5%)	46 (37.1%)	21 (43.7%)	
Pathological stage				0.002
Ⅰ	220 (88.4%)	103 (83.1%)	33 (68.8%)	
Ⅱ	14 (5.6%)	16 (12.9%)	6 (12.5%)	
Ⅲ	15 (6.0%)	5 (4.0%)	9 (18.8%)	
Adjuvant chemotherapy				0.773
No	220 (88.4%)	107 (86.3%)	41 (85.4%)	
Yes	29 (11.6%)	17 (13.7%)	7 (14.6%)	
Recurrence				< 0.001
No	211 (84.7%)	98 (79.0%)	29 (60.4%)	
Yes	38 (15.3%)	26 (21.0%)	19 (39.6%)	

### 2.2 肺功能情况

随COPD严重程度的增加，FVC、FVC%、FEV_1_、FEV_1_%、FEV_1_/FVC%均降低，差异有统计学意义（*P* < 0.05，表 2）；FEV_1_%与其他肺功能指标FVC、FVC%、FEV_1_、FEV_1_/FVC%均有相关性（*P* < 0.001），表明基于FEV_1_%值的COPD严重程度分级可以准确反映患者肺功能情况。

**2:** COPD严重程度不同的NSCLC患者肺功能情况（Mean±SD） Comparison of pulmonary function among groups with different severity of COPD (Mean±SD)

Pulmonary function parameter	Non-COPD (*n*=249)	Mild COPD (*n*=124)	Moderate/severe COPD (*n*=48)	*P*
FVC (L)	3.30±0.84	3.08±0.74	3.02±0.81	0.022
%FVC (%)	113.35±15.97	107.19±17.38	97.14±17.46	< 0.001
FEV_1_ (L)	2.21±0.77	1.90±0.72	1.58±0.53	< 0.001
%FEV_1_ (%)	109.61±16.68	82.93±18.50	69.93±9.44	0.045
FEV_1_/FVC (%)	78.04±6.32	63.39±5.43	54.41±9.20	< 0.001

### 2.3 预后因素分析

总体1年、3年、5年无复发生存率分别为96.7%、85.2%、72.7%（图 1）。伴COPD的NSCLC患者5年无复发生存率为64.0%，低于不伴COPD者（78.1%），差异有统计学意义（*P*=0.002，图 2）；亚组分层分析结果显示，无COPD组、轻度COPD组的5年无复发生存率分别为78.1%和70.4%，差异无统计学意义（*P*=0.115），中重度COPD组5年无复发生存率为46.4%，与无COPD组或轻度COPD组之间差异均有统计学意义（*P* < 0.001，*P*=0.001，图 3）。单因素*Kaplan-Meier*生存分析显示，年龄、性别、吸烟史、COPD严重程度、肿瘤直径、组织学类型和病理分期是患者预后的影响因素（表 3）。将单因素分析中有统计学意义的变量纳入*Cox*比例风险模型，多因素分析结果显示，年龄、性别、中重度COPD和病理分期是影响患者预后的独立危险因素，COPD严重程度高危者预后差（HR=1.718, 95%CI: 1.086-2.919）（表 4）。

**1:**
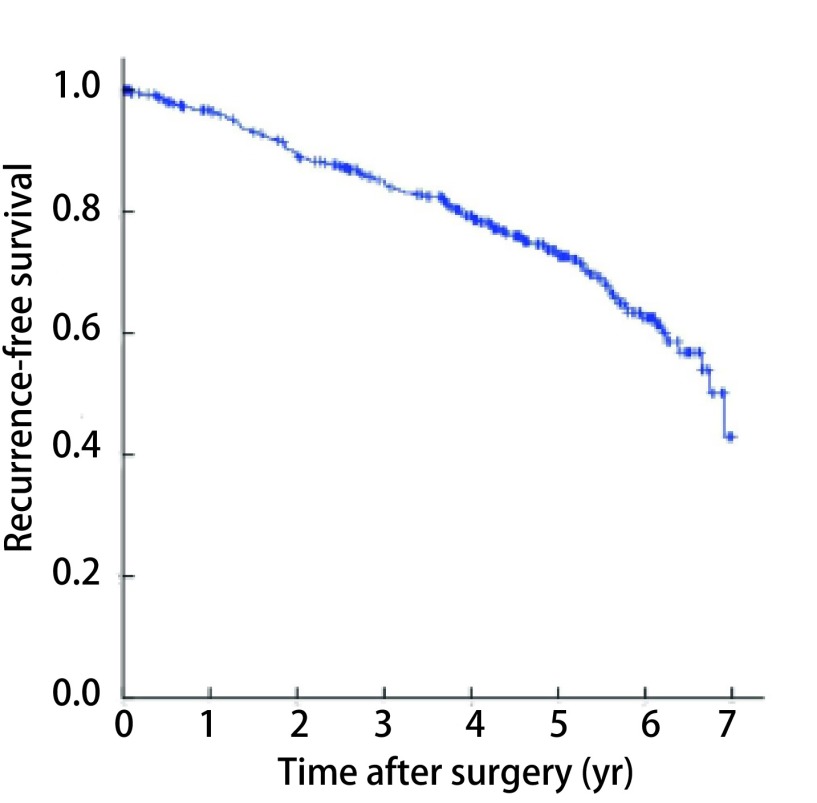
全组无复发生存曲线 *Kaplan-Meier* survival curves of recurrence-free survival for the whole study population

**2:**
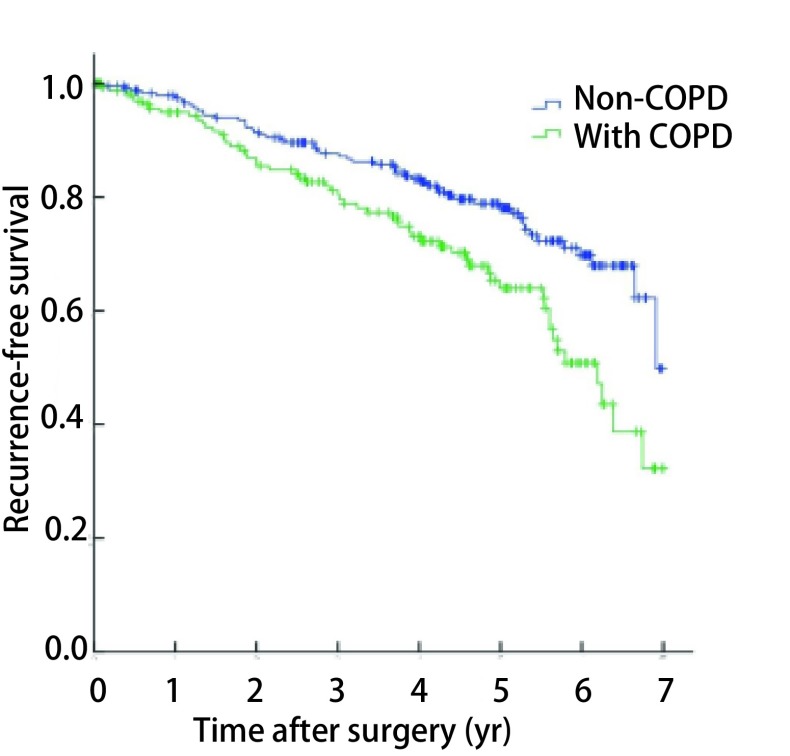
无COPD的NSCLC患者与伴COPD者无复发生存曲线比较（*P*=0.002） Kaplan-Meier survival curves for patients with NSCLC according to the presence of COPD (*P*=0.002)

**3:**
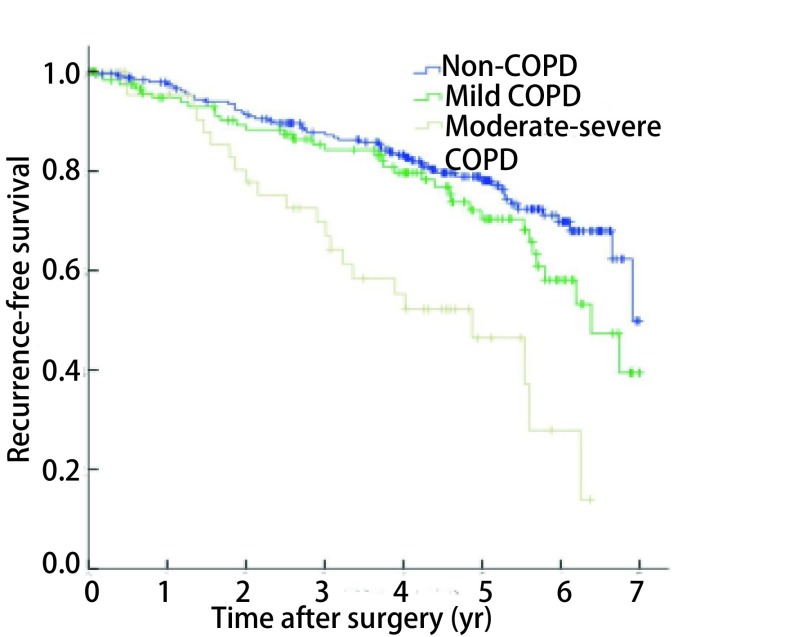
COPD严重程度不同的NSCLC患者无复发生存曲线比较（*P* < 0.001） *Kaplan-Meier* survival curves for patients with NSCLC according to COPD severity (*P* < 0.001)

**3:** 患者预后影响因素的单因素分析结果 Univariate analyses to identify factors associated with recurrence-free survival

Factor	Cases (*n*=421)	5-yr RFS rate (%)	*P*
Age (yr)			0.001
< 65	155	82.7	
≥65	266	65.4	
Gender			< 0.001
Female	161	85.9	
Male	260	63.8	
Smoking history			< 0.001
No	148	82.8	
Yes	273	66.7	
COPD			< 0.001
Non	249	78.1	
Mild	124	70.4	
Moderate/Severe	48	46.4	
Tumor size (cm)			0.002
≤3	355	74.8	
> 3	66	58.5	
Tumor location			0.372
Left upper lobe	114	69.6	
Left lower lobe	61	68.5	
Right upper lobe	132	78.4	
Right middle lobe	29	79.2	
Right lower lobe	85	68.2	
Histology			< 0.001
Adenocarcinoma	318	77.3	
Non-adenocarcinoma	103	56.9	
Pathological stage			< 0.001
Ⅰ	356	78.4	
Ⅱ	36	54.3	
Ⅲ	29	43.2	
Adjuvant chemotherapy			0.351
No	368	74.4	
Yes	53	68.3	
RFS: recurrence-free survival.			

**4:** 患者预后影响因素的*Cox*多因素分析结果 Multivariate analysis of risk factors for recurrence-free survival with proportional hazard model

Factor	B	SE	Wald *χ*^2^	Hazard ratio	95%CI	*P*
Age (≥65 *vs* < 65)	0.630	0.216	8.496	1.878	1.229-2.868	0.004
Gender (male *vs* female)	0.720	0.285	6.394	2.055	1.176-3.593	0.011
Smoking history (Yes *vs* No)	0.260	0.290	0.799	1.296	0.734-2.290	0.371
Moderate/severe COPD (Yes *vs* No)	0.577	0.252	5.234	1.718	1.086-2.919	0.022
Tumor size ( > 3 *vs* ≤3)	0.305	0.223	1.333	1.357	0.808-2.277	0.248
Histology (Non-Ad *vs* Ad)	0.255	0.220	1.334	1.290	0.837-1.987	0.248
Pathological stage (Ⅱ/Ⅲ *vs* Ⅰ)	1.213	0.264	29.509	3.363	2.171-5.210	< 0.001

## 3 讨论

慢性炎症在肺癌的发生和发展中起到重要作用^[6]^，COPD是呼吸系统最常见的慢性炎症性疾病，临床中常见肺癌合并COPD的患者，因此推测肺癌与COPD可能存在复杂的内在联系^[7]^。COPD和肺癌两者在发病危险因素方面存在重叠，如吸烟和环境因素与两者关系密切。每年约有1.67%的COPD患者发展成为肺癌，约有20%-30%的COPD患者死于肺癌^[3, 4]^。一项纳入3, 371例患者的研究结果显示，COPD患者发生肺癌的危险性是对照组的2.06倍，发生风险随着慢阻肺严重程度加重而显著增高，COPD是肺癌发病的独立危险因素^[8]^。国外研究报道，临床上新诊断的肺癌患者进行肺功能测定，可发现40%-70%的肺癌患者同时合并存在COPD^[4, 9]^。但由于在FEV_1_显著下降前患者一般没有症状，因此在肺癌患者中有72%-93%的COPD未被诊断^[10-12]^。本组患者中符合COPD诊断标准者占40.8%，有吸烟史者占64.8%，与文献报道一致。因肺叶切除术对肺功能有严格的要求，本组患者COPD严重程度以轻、中度为主，仅2例重度，无极重度COPD者。COPD严重程度与高龄、男性、吸烟、非腺癌有关，本组数据表明FEV_1_%与其他肺功能指标FVC、FVC%、FEV_1_、FEV_1_/FVC%均有相关性，FEV_1_%可代表患者肺功能情况，基于FEV_1_%值的COPD严重程度分级可以很好区分不同肺功能状态的NSCLC。

COPD严重程度与肺切除术后心肺并发症发生率相关已被大量研究证实，肺功能减退者术后心肺并发症高于肺功能正常者，生存质量较肺功能正常者差^[13]^。但COPD对肺癌根治术后远期生存的影响仍存在争议，不同的研究结果甚至相冲突。一些研究^[14]^认为伴COPD的NSCLC预后不佳，Gullón等^[15]^发现肺气肿是影响NSCLC患者生存的一个不良预后因素；Kiri等^[16]^报道，合并COPD的肺癌患者3年生存率仅为单纯肺癌患者的一半；Sekine等^[17]^报道COPD是Ia期肺癌完全切除术后复发的危险因素，伴COPD者5年生存率为77.0%，不伴COPD者5年生存率为91.6%，差异有统计学意义（*P*=0.000, 1）。但与此相反的是，Arca等^[18]^认为伴COPD的患者生存期更长，而另两个研究却发现是否伴COPD与NSCLC的预后并没有相关性^[19, 20]^。

出现这些差异的原因可能与不同研究的入组标准、样本量、疾病分期、治疗方式等因素不同有关。因此本研究中将观察对象限定为接受胸腔镜肺叶切除术、系统性纵隔淋巴结清扫的Ⅰ期-Ⅲa期NSCLC患者，并未纳入开胸手术及亚肺叶切除、复合肺叶切除或全肺切除者，以减少术式因素的影响。本研究结果表明，随着COPD严重程度增加，病理分期和术后复发率增加，伴COPD的NSCLC患者术后5年无复发生存率显著低于不伴COPD者。进一步的亚组分析表明，中重度COPD（FEV_1_% < 80%预计值）是造成该结果的主要原因，也是Cox回归多因素分析中独立的预后危险因素，而轻度COPD（FEV_1_≥80%预计值）并未对无复发生存有显著影响。

COPD影响NSCLC预后可能的机制是：在COPD炎性肺组织中发生的肺癌侵袭性更高^[2]^，炎性细胞因子、趋化因子产生的活化作用能促进肺癌的发生发展，核因子-κB（nuclear-factor kappa B, NF-κB）、转录信号转导子与激活子3（signal transducers and activators of transcription 3, STAT3）等炎症反应的关键转录因子与肺癌的发生、转移有关，可以促进肿瘤细胞增殖，参与肿瘤血管的生成，促进上皮-间质细胞转化（epithelial-mesenchymal transition, EMT），进而促进肿瘤侵袭和转移。COPD患者肺组织中基质金属蛋白酶、细胞黏附因子-1表达增加，引起肺组织破坏和重塑、降解细胞外基质和基底膜，有利于肿瘤的侵袭和转移^[21]^。

本研究说明合并中重度COPD是影响NSCLC患者术后无复发生存的独立危险因素，可结合患者术前肺功能来判断预后，更准确地预测复发风险，为高危患者制定合理的个体化治疗方案。作为单中心的回顾性研究，结论是否有普遍意义，有待更多的临床研究进一步验证。并且在将来的基础研究中，可以从慢性炎症出发探讨炎症通路和免疫状态对肺癌的影响，对明确肺癌的发病机制、寻找新的治疗靶点和改善预后有重要意义。
